# Rapid Detection of *bla*_KPC-9_ Allele from Clinical Isolates

**DOI:** 10.3390/pathogens10040487

**Published:** 2021-04-17

**Authors:** Konstantina Gartzonika, Petros Bozidis, Ephthalia Priavali, Hercules Sakkas

**Affiliations:** 1 Microbiology Department, Faculty of Medicine, School of Health Sciences, University of Ioannina, 45110 Ioannina, Greece; pbozidis@uoi.gr (P.B.); isakkas@uoi.gr (H.S.); 2 Microbiology Department, University Hospital of Ioannina, 45110 Ioannina, Greece; epriaval@cc.uoi.gr

**Keywords:** *K. pneumoniae*, carbapenemases, *bla*_KPC-2_, *bla*_KPC-9_, RsaI, ARMS, outbreak

## Abstract

The emergence of *Klebsiella pneumoniae* carbapenemase (KPC) nosocomial outbreaks related to specific *bla*_KPC_ gene variants dictates the need for applicable diagnostic methods for allele discrimination. We report here a simple method of *bla*_KPC-9_ allele recognition based on a combination of endonuclease digestion analysis and PCR amplification using unique primers. *K. pneumoniae* isolates carrying the *bla*_KPC_ gene were tested. Digestion with RsaI restriction endonuclease was found to efficiently differentiate the *bla*_KPC-2_ from the *bla*_KPC-9_ variants into two distinct groups of digestion patterns named KPC-2-like and KPC-9-like, respectively. An additional procedure, the amplification refractory mutation system (ARMS) method, was applied to identify the variant within the same group. The principles of this procedure could be developed to identify several *bla*_KPC_ gene variants, as well as monitoring the spread and evolution of specific KPC variants within local geographical regions.

## 1. Introduction

*Klebsiella pneumoniae* carbapenemase-possessing *K. pneumoniae* (KPC-Kp) clinical isolates have been implicated in hospital outbreaks in different geographical regions, including Greece [1]. Several amino acid sequence variants of KPC have been described, suggesting the continued evolution of resistance in the KPC-2 enzyme, which is the most prevalent variant worldwide [2]. To date, the nucleotide sequences of 82 KPC gene variants have been deposited in the National Center for Biotechnology Information (NCBI, U.S. National Library of Medicine) databases [3]. The presence of KPC-Kp strains was reported in Greece in 2008 [4], and since then, epidemiological studies conducted in hospitals throughout the country reported *K. pneumoniae* strains belonging to 11 sequence types [5], harboring mainly the KPC-2 variant [6] and in some sporadic cases the KPC-3 [6] and KPC-23 [7] variants. Only recently were *K. pneumoniae* strains carrying a different *bla*_KPC_ gene variant, the KPC-9 variant, identified among KPC-2 producing isolates of an ongoing outbreak taking place in a tertiary hospital in the Northwestern part of Greece [8].

The emergence of KPC nosocomial outbreaks related to these specific *bla*_KPC_ gene variants within the Greek territory, dictates the need for applicable diagnostic methods for allele discrimination. Prompt and accurate recognition of KPC variants is critical to identify transmission pathways, especially in cases of highly virulent or resistant strains which may require the implementation of specific infection control measures to restrict their dissemination. Although *bla*_KPC_ gene direct sequencing or whole-genome sequencing (WGS) are the most appropriate methods for *K. pneumoniae* subtyping, they are both time-consuming procedures, requiring specialized equipment and personnel. We here report a simple method based on a combination of polymerase chain reaction (PCR) amplification using unique primers and endonuclease digestion analysis that could provide a safe shortcut in the positive discrimination of the *bla*_KPC-9_ alleles in clinical samples. Moreover, the principles of this method could be applied to the identification of more *bla*_KPC_ gene variants.

## 2. Results

### 2.1. Molecular Testing and RsaI Restriction Mapping of bla_KPC_ Gene Variants

**Figure 1:**
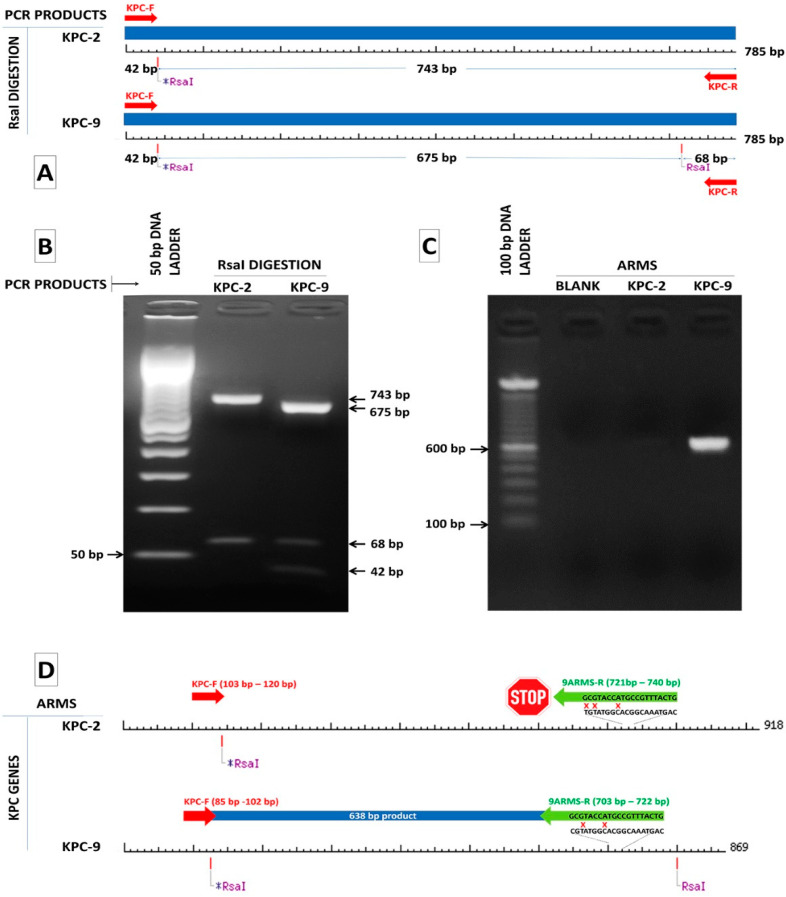
(**A**) RsaI digestion pattern of the 785 bp KPC-2 and KPC-9 amplicons where RsaI sites and lengths of the generated fragments are indicated. (**B**) Gel electrophoresis of RsaI digested amplicons of KPC-2 and KPC-9 positive samples confirming the production of the anticipated fragments by digestion. (**C**) Design of a reverse ARMS specific primer for the discrimination of the KPC-9 allele from the KPC-2 allele. (**D**) A 638 bp product is produced only in the case of a KPC-9 positive sample.

During the study period, 181 *K. pneumoniae* isolates were phenotypically shown to produce class A carbapenemases. The *bla*_KPC_ carriage was verified by conventional PCR assay. Based on RsaI endonuclease digestion, two different digestion patterns were identified, including the PCR amplicons with one and two restriction sites for RsaI, respectively (Figure 1A,B). The PCR templates of 47 out of 181 clinical isolates were digested, producing three restriction fragments presenting the same electrophoretic profile.

**Figure 2:**
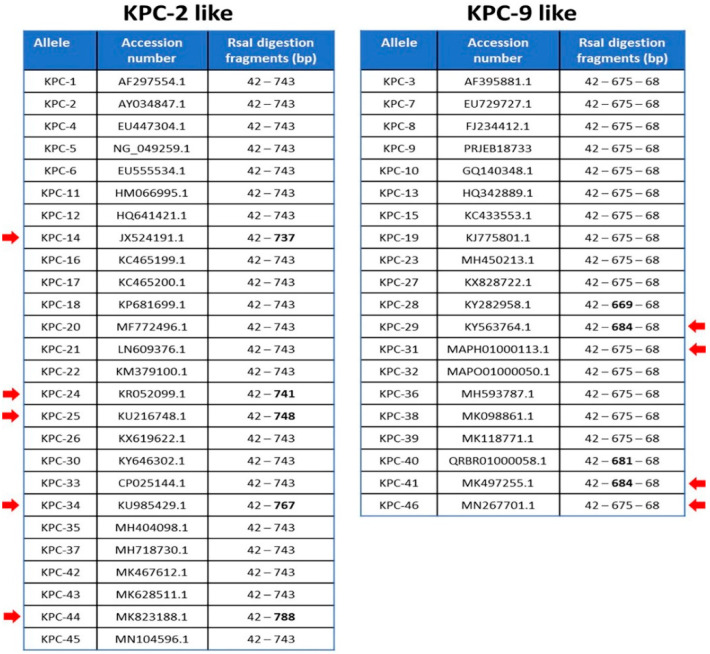
The amplicons generated by KPC corresponding variants are separated into two groups according to RsaI digestion pattern (KPC-2-like and KPC-9-like). The anticipated length of digestion fragments for each amplicon is indicated along with the accession number of the reference sequence for each KPC variant. Red arrows dictate the fragments of variable length.

We also selected 46 alleles of *bla*_KPC_ genes reporting to NCBI database in order to categorize them based on RsaI digestion. The nucleotide sequences of 37 out of 46 alleles (*bla*_KPC-(1–4)_, *bla*_KPC-(6–9)_, *bla*_KPC__-(11–12)_, *bla*_KPC-(14–17)_, *bla*_KPC-19_, *bla*_KPC-(22–27)_, *bla*_KPC-(29–39)_, *bla*_KPC-(41–44)_ and *bla*_KPC-46_) have been isolated from *K. pneumoniae* strains, four (*bla*_KPC-18_, *bla*_KPC-(20–21)_ and *bla*_KPC-28_) from *Escherichia coli* strains, two (*bla*_KPC-13_ and *bla*_KPC-45_) from *Enterobacter cloacae* strains and one (*bla*_KPC-40_, *bla*_KPC-5_, *bla*_KPC-10_) from *Enterobacter hormoechei, Pseudomonas aeruginosa* and *Acinetobacter baumannii* strains, respectively. As it is depicted, in Figure 2, all amplicons have at least one and not more than two RsaI restriction sites. The two KPC-variants of interest, KPC-2 and KPC-9, belong to two different groups of digestion patterns, named KPC-2-like and KPC-9-like after the two corresponding variants. The KPC-2 amplicon of 785 bp was digested by RsaI endonuclease in two fragments of 42 bp and 743 bp, while the KPC-9 amplicon of the same length was digested in three fragments of 42 bp, 675 bp and 68 bp (Figure 1A,B). All examined alleles could also be categorized into the two aforementioned distinct groups by RsaI digestion (Figure 2). According to that, 47 clinical isolates classified to the KPC-9-like group and the remaining 134 to the KPC-2-like group.

Twelve archived *K. pneumoniae* strains previously characterized by WGS were used as control strains; eight were identified to carry the KPC-9 variant and four the KPC-2 variant [8]. In all cases, the PCR amplification and the downstream RsaI digestion of the amplified sequences generated the anticipated fragments (Figure 1B). All the examined strains were distributed in the two KPC-like groups in accordance with the original characterization by WGS.

### 2.2. KPC-9 Identification by Amplification Refractory Mutation System (ARMS) Method

**Figure 3:**
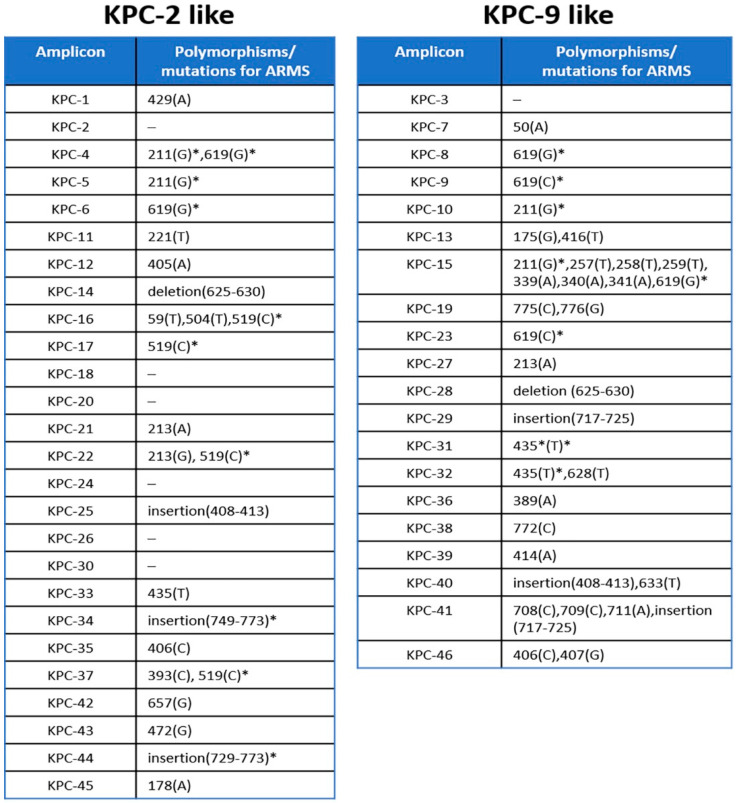
Nucleotide positions of polymorphisms or mutations in KPC variants amplicons that could be used for the implementation of ARMS discrimination within each group. * Positions marked by asterisk denote polymorphisms that appear in more than one sequence and thus the discrimination of the corresponding amplicons should be based on the combined detection of the rest of the substitutions.

Since the KPC-9 allele could be properly distinguished from the KPC-2-like alleles, we further investigated the possibility of its discrimination from the alleles of the same group. To this end, we used a PCR-based assay, the ARMS method, which is able to detect single nucleotide polymorphisms among homolog sequences based on the design of specific primers targeting these polymorphisms. The nucleotide comparison of the sequences deposited in the NCBI Genbank genetic sequence database (https://www.ncbi.nlm.nih.gov/genbank/) (accessed on 9 March 2020) for the selected variants, belonging to KPC-9-like group, showed that there is at least one nucleotide polymorphism in each sequence (Figure 3).

The only exception is the KPC-3 sequence, which does not have any substitutions that compare to the rest variants of the KPC-9-like group (data not shown). The comparison also revealed that the KPC-9 and KPC-23 sequences (PRJEB18733 and MH450213.1, respectively) have 100% sequence identity and can allow for the design of a specific ARMS primer targeting a cytosine (C) at position 721 of the KPC-9 sequence, which substitutes for thymidine (T) in the rest of KPC-9-like sequences. A KPC-R-9-arms specific primer was designed, having its 3′end complementary to the cytosine 721 and two mismatches upstream of its 3′end in order to enhance the specificity of binding (Figure 1C). The efficiency of ARMS method to amplifying only the KPC-9 sequence was tested in the same 12-well characterized control strains of *K. pneumoniae* carrying the KPC-2 or KPC-9 alleles. A 683 bp amplicon was generated by the KPC-R-9-arms specific primer in both KPC-9 control strains and 47 clinical isolates belonging to the KPC-9-like group. On the other hand, no amplicons were observed for any other strains (Figure 1D). The ARMS-PCR assay was 100% concordant with results retrieved from the sequencing of PCR products.

## 3. Discussion

The global dissemination of KPC-producing *K. pneumoniae* seems to be an emerging public health problem in various parts of the world [9,10]. Predominantly KPC-Kp strains have so far been identified in Greece [1]. Most of the strains that are related to outbreaks have been found to carry the KPC-2 allele and only recently did the KPC-9 allele make its appearance in an outbreak among KPC-Kp strains [8]. The phenotypic discrimination of KPC alleles is not possible, since they show no differences regarding the patterns of antimicrobial resistance. However, the KPC variants are not equal and have different susceptibilities and hydrolytic properties, as is the case for KPC-5, which leads to high-level cefixime resistance [11]. Some other variants that possess point mutations also exhibit increased catalytic efficiency for ceftazidime hydrolysis, compared with KPC-2, leading to an increase in the minimal inhibitory concentration (MIC) for ceftazidime [2,11,12]. The discrimination of KPC variants is not of such importance for the selection of antimicrobial therapy but is mandatory because of their potential to spread and evolve in response to local selective pressures [13].

**Figure 4:**
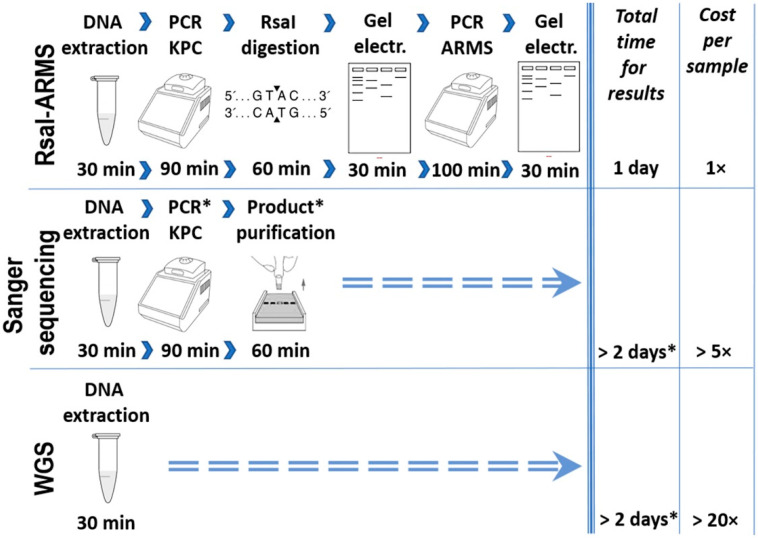
A comparative workflow for RsaI-ARMS, Sanger sequencing and WGS for *bla*_KPC_ allele identification which summarizes the estimated gains of using the RsaI-ARMS method. * Procedures or time frames denoted with asterisks refer to clinical laboratories which are not capable of performing Sanger sequencing or WGS in their facilities.

We propose here a simple method of KPC allele recognition which is divided into two steps. This two-step method for *bla*_KPC_ variant identification shows certain advantages against Sanger sequencing and WGS in terms of both time and financial cost (Figure 4). Although the other two techniques may be considered as the “gold standard” for allele identification, they require facilities and trained personnel, which most of the clinical laboratories do not possess. In our study, the first step is designed to separate the amplicons of all known variants into two groups, based on the difference in digestion pattern. Digestion with RsaI restriction endonuclease has been reported to effectively differentiate the *bla*_KPC-2_ from the *bla*_KPC-3_ variant [14]. We proved that this procedure is efficient to separate the amplicons of all the known variants that were analyzed, into two distinct groups. During the second step, the ARMS method could be applied to identify the variant within the same group. ARMS PCR was successfully used to identify strains which harbor a variant, the IMP-6 [15], or to distinguish between carbapenemase-type *bla*_GES_ and ESBL-type *bla*_GES_ without sequencing [16]. In our study, nucleotide comparison among all amplicons of the KPC-2-like and KPC-9-like groups showed that the ARMS method could also be used in the identification of most of them. Figure 3 includes all the positions of the nucleotide polymorphisms and the mutations that can be used for the method and reveals the limitations for the implementation of the method. More specifically, all the amplicons of the KPC-2-like group could be identified besides the ones generated by *bla*_KPC-2_, *bla*_KPC-18_, *bla*_KPC-20_, *bla*_KPC-24_, *bla*_KPC-26_, and *bla*_KPC-30_ variants. Although these amplicons may be identical, the nucleotide comparison of their complete sequence showed that they bear single nucleotide polymorphisms upstream of the amplified sequence, which could be used for their discrimination in an analogous way (data not shown).

Correspondingly, all the amplicons within the KPC-9-like group besides the one generated by the *bla*_KPC-3_ variant, could be identified by ARMS. This variant does not bear any polymorphisms either within the amplified sequence nor within the sequences upstream and downstream of the amplicon. Furthermore, the mutations marked with an asterisk appear in more than one variant and thus these variants could be discriminated in combination. In addition, KPC-23 appears to be the complete version of KPC-9 [7]. The nucleotide comparison of *bla*_KPC-9_ and *bla*_KPC-23_ complete sequences showed that they essentially share the same sequence aside from an additional region of 13 nt directly upstream of the 5′ end of *bla*_KPC-9_ appearing in a *bla*_KPC-23_ reference sequence. It should also be noted that the NCBI Genbank database’s reference sequence for KPC-1 (AF297554.1) bears a nucleotide substitution in position 650 (A to G) compared to the KPC-2 reference sequence AY034847.1 (position 525), although these sequences are now, after a published correction of the KPC-1 sequence [17], considered to be the same. Until now, KPC-2 and KPC-9 variants have been recognized in our region and we succeeded in discriminating them based on this method. Further studies focusing on the characteristics and comparative analysis of the virulence of these variants, should be conducted.

In conclusion, the ARMS methodology, combined with RsaI digestion, (1) enabled us to accurately and quickly identify KPC-9-producing *K. pneumoniae* isolates; (2) could be extended for the discrimination of the most KPC variants; (3) could be useful in cases in which subtyping through direct sequencing is not feasible for monitoring or investigating the spread and evolution of specific KPC variants within local geographical regions.

## 4. Materials and Methods

### 4.1. Bacterial Isolates, Screening for KPC Carbapenemase

This study was initiated as part of an outbreak investigation after the randomly identification of KPC-9-Kp isolated from patients staying at the same ward of the University Hospital of North-western Greece [8]. Following initial KPC-9-Kp identification, all *K. pneumoniae* strains isolated from inpatients during a two-year period, were analysed. Isolates were previously characterized using a VITEK 2 automated system (bioMerieux, Marcy l’Etoile, France). Screening for class A and B carbapenemases was performed in isolates displaying resistance or reduced susceptibility, based on the Clinical and Laboratory Standards Institute (CLSI) criteria, to one or more of carbapenems [4]. Phenotypically suspected KPC isolates were further investigated for the presence of the KPC gene. DNA was extracted using the QIAamp DNA Mini Kit (QIAGEN Gmbh, Hilden, Germany) according to the manufacturer’s instructions. PCR reaction was performed in a final volume of 50 μL containing 100 ng of total DNA, 1× HotStarTaq PCR buffer (Tris pH 8.7, KCl, (NH_4_)_2_SO_4_, 1.5 mM MgCl_2_), 200 μM dNTP, 40 pmol of each of the KPC-F-Universal (5′-TCGCTAAACTCGAACAGG-3′) and KPC-R-Universal (5′-TTACTGCCCGTTGACGCCCAATCC-3′) universal primers targeting the *bla*_KPC_ alleles [18] and 1 U of HotStarTaq DNA polymerase (Qiagen, Gmbh, Hilden, Germany). A conventional thermocycler (PTC-200, Pelter Thermal Cycler, MJ Research, Inc., Waltham, Massachusetts, USA) was used for amplification, which consisted in an initial denaturation at 94 °C for 10 min followed by 36 cycles of 94 °C for 30 s, 52 °C for 40 s and 72 °C for 50 s with a final extension at 72 °C for 10 min. PCR products were analyzed by electrophoresis on a 2% (*wt*/*vol*) agarose gel in 1× TBE buffer stained with ethidium bromide (10 μM) and visualized under UV light. The size of each amplicon was estimated using a 100 bp DNA ladder (Invitrogen, ThermoFisher Scientific Baltics UAB, Vilnius, Lithuania). The products of interest were excised from the gel, purified with the QIAquick Gel Extraction kit (Qiagen, Gmbh, Hilden, Germany) and sequenced with the Sanger method on an ABI PRISM 3130 Genetic Analyzer (Applied Biosystems Japan Ltd., Tokyo, Japan). The nucleotide sequences were analysed using the Basic Local Alignment Search Tool (BLAST, vs. 2.10.1).

### 4.2. Restriction Fragment

Following the PCR detection of *bla*_KPC_, amplicons were digested with Thermo Scientific RsaI restriction enzyme (10 U/μL, Invitrogen™, Thermo Fisher Scientific Inc., Waltham, MA, USA) using NEBcutter V2.0 (New England Biolabs Inc., Ipswich, MA, USA). RsaI enzyme was selected based on the published sequences for the KPC variants in the NCBI nucleotide database (www.ncbi.nlm.nih.gov/nuccore/?term) (accessed on 9 March 2020). In total, 10 μL of the PCR product was digested in a final volume of 20 μL using 5 U of RsaI enzyme at 37 °C for 1 h, and the digested fragments were analyzed by agarose gel electrophoresis, as mentioned above. The RsaI digestion pattern was extended in most nucleotide sequences reported to NCBI database that have been correlated to *bla*_KPC_ variants. *K. pneumoniae* clinical strains known to possess the *bla*_KPC-9_ gene were also tested to evaluate the effectiveness of this assay.

### 4.3. KPC-9 PCR Using Specific Primer

In order to distinguish the KPC-9 allele from other KPC alleles, a PCR-based technique, the ARMS method, was used [19,20]. The KPC-R-9-arms specific primer (5′-GTCATTTGCCGTACCATGCG-3′) was designed using Primer3web software (v.4.1.0) (https://bioinfo.ut.ee/primer3/) (accessed on 21 May 2020). Additional mismatches were incorporated at the 3′end of the KPC-R-9-arms specific primer in order to enhance the discrimination between the KPC-9 and KPC-2 alleles [19]. An amplicon of 638 bp was produced only in the presence of the KPC-9 allele in the sample. PCR-ARMS reactions for each allele, were performed in a final volume of 25 μL containing 50 ng total DNA, 1X PCR buffer, 2.5 mM MgCl_2_, 200 μM dNTP, 0.25 μM ARMS primer, 0.25 μΜ KPC-F-Universal primer and 2.5 U Taq DNA polymerase (Hytest Ltd., Turku, Finland). The amplification reaction was performed in a conventional thermocycler (PTC-200, Pelter Thermal Cycler, MJ Research, Inc., Waltham, Massachusetts, USA) using the following conditions: 95 °C for 5 min followed by 35 cycles of 94 °C for 45 s, 55 °C for 45 s and 72 °C for 1 min and a final extension step at 72 °C for 10 min. The PCR products were analyzed by electrophoresis and visualized as mentioned above. Discrimination between the KPC-2 group and KPC-9 isolates was determined by the absence or presence, respectively, of the amplified DNA products in electrophoresis.

## Data Availability

The data presented in this study are available in this article.
